# Association between Single Nucleotide Polymorphisms (SNPs) and Toxicity of Advanced Non-Small-Cell Lung Cancer Patients Treated with Chemotherapy

**DOI:** 10.1371/journal.pone.0048350

**Published:** 2012-10-31

**Authors:** Ling Zhang, Guanghui Gao, Xuefei Li, Shengxiang Ren, Aiwu Li, Jianfang Xu, Jie Zhang, Caicun Zhou

**Affiliations:** Department of Oncology, Shanghai Pulmonary Hospital, Tongji University School of Medicine, Tongji University Cancer Institute, Shanghai, China; University Clinic of Navarra, Spain

## Abstract

New therapeutic approaches are being developed based on the findings that several genetic abnormalities underlying non-small-cell lung cancer (NSCLC) could influence chemosensitivity. In this study, we assessed whether polymorphisms in genes of nucleotide excision repair (NER) pathway, including ERCC5, ERCC6, MMS19L, CCNH, XPC, RRM1, can affect the tolerability of platinum-based chemotherapy in NSCLC patients. We used AllGloTM probe to assess genotyping and polymorphisms in 388 stage IIIB and IV NSCLC patients treated with platinum-based chemotherapy. MMS19L might be associated with the adverse events of chemotherapy in NSCLC, especially for all grade leucopenia (*P* = 0.020), all grade jaundice (*P* = 0.037) and all grade creatinine increasing (*P* = 0.013). In terms of grade 3/4 adverse events, MMS19L was related with total grade 3/4 adverse events (*P* = 0.024) and grade 3/4 thrombocytopenia (*P* = 0.035), while RRM1 was related with total grade 3/4 adverse events (*P* = 0.047) and grade 3/4 vomiting (*P* = 0.046). ERCC5 was related with more infection (*P* = 0.017). We found that some SNPs in NER pathway genes were correlated with toxicity treated with double chemotherapy in advanced NSCLC patients, especially for SNPs of MMS19L, RRM1 and ERCC5.

## Introduction

Non-small-cell lung cancer (NSCLC) is the leading cause of cancer related deaths worldwide [1]. Platinum based doublet chemotherapy with any third generation cytotoxic agent (vinorelbine, gemcitabine, paclitaxel, docetaxel, pemetrexed), has improved the survival of advanced NSCLC (stage IIIB or IV). When compared head-to-head in phase III studies, these doublets have shown comparable efficacy, with differences in toxicity.

A total of 1207 patients enrolled in the ECOG 1594 study, the results indicated that 85 to 93 percent of patients developed grade 3 to 5 toxicity, in the 1183 evaluable patients. The common adverse events of combination chemotherapy were myelosuppression, infection, febrile neutropenia, cardiac toxicity, renal dysfunction, nausea/vomiting, diarrhea, hypersensitivity, weakness, etc. 15 to 27 percent of patients were withdrawn from the chemotherapy because of side effects [2]. A total of 1725 patients enrolled in the JMDB (pemetrexed plus cisplatin versus gemcitabine plus cisplatin) phase III study. Hematological grade 3/4 drug-related toxicities (including neutropenia, anemia and thrombocytopenia) were 25 percent and 50 percent, respectively [3]. 39.9 to 48 percent patients experienced grade 3/4 adverse events in TAX 326 study, which enrolled 1218 patients to compare docetaxel plus platinum versus vinorelbine plus cisplatin for advanced NSCLC as first line treatment [4].

Treatment-related toxicity may impact the efficacy of chemotherapy by decreasing the dose-density and dose-intensity, which resulted in therapy holding, dose modification or omitting. Lethal chemotherapy toxicities are not frequent but do happen in our daily practice [5]. Although cytotoxic chemotherapy improves survival compared with best supportive care in advanced NSCLC, its advantage may be hampered by clinically relevant toxicities [6].

With regard to toxicity, polymorphisms in the drug-metabolizing enzymes are found to be associated with the inter-individual variation in response to a particular drug [7]. Nucleotide-excision repair (NER) represents a pathway involved in detection and repair of DNA base damage, most notably those caused by environmental exposures such as chemical exposures, defends against cytotoxicity [8].

The use of germline genetic variants such as single nucleotide polymorphisms (SNPs) is an alternative and complementary approach and has produced promising results [9]. Nucleotide excision repair (NER) is the primary DNA repair pathway responsible for the removal of cisplatin-DNA adducts. Other cisplatin-related pathways include drug uptake, metabolism, and efflux, regulation of cell cycle checkpoints, and apoptosis [8].

Several studies have found some SNPs of NER genes, such as ERCC1, XRCC1, XPD and MDR1, may be related with severe toxicity in stage III and IV NSCLC patients treated with chemotherapy [10]–[12]. But conferred risk of chemotherapy of NSCLC by SNPs in the NER pathway has not been definitively answered. Many other SNPs, including ERCC5, ERCC6, MMS19L, CCNH, XPC and RRM1 related with DNA damage and repair, may play a important role in treatment-related toxicity. In this study, we assessed whether polymorphisms in genes of NER pathway, including ERCC5, ERCC6, MMS19L, CCNH, XPC and RRM1, are associated with affecting the tolerability of platinum-based chemotherapy in stage IIIB and IV NSCLC patients. We have found some SNPs of NER genes may be related with severe toxicity in advanced NSCLC patients treated with platinum-based chemotherapy.

## Materials and Methods

### Subjects

Patients were chemo-naive staged IIIB or IV NSCLC, with histological or cytological confirmed. Briefly, patients had an Eastern Cooperative Oncology Group (ECOG) performance status (PS) of 0 to 1 and adequate bone marrow reserve and organ function, such as hemoglobin >9 g/dl, neutrophil count >1.5×10^9^/L, and platelet count ≥100×10^9^/L, renal function (creatinine clearance rate >50 ml/s) and liver function (bilirubin <1.5 times the normal upper limit, aspartate aminotransferase and alanine aminotransferase <2.5 times the normal upper limit), aged over 18 years.

Patients were excluded from the study for symptomatic brain metastases, spinal cord compression, uncontrolled massive pleural or pericadial effusion, and previous chemotherapy. The protocol was conducted according to the Declaration of Helsinki and Good Clinical Practice guidelines and was approved by the ethics committees of Tongji University Affiliated Shanghai Pulmonary Hospital. The informed consent was written and obtained from each patient before the initiation of any study related procedure. The protocol was conducted in Shanghai Pulmonary Hospital. This was a prospectively collection of clinical data and biological samples and later the study was designed. Enrollment started in October 2005 and ended in March 2009.

### Treatment and Clinical Assessments

Patients received intravenous doses of vinorelbine 25 mg/m^2^ or gemcitabine 1000 mg/m^2^ on day 1 and 8, or docetaxel 75 mg/m^2^ or paclitaxel 175 mg/m^2^ on day 1 plus cisplatin 75 mg/m^2^ or carboplatin AUC = 5 on day 1, every 21 days per cycle, for a maximum of six cycles, up to disease progression or unacceptable toxicity. Chemotherapy was given only with a neutrophil count of >1.5×10^9^/L, a platelet count of ≥100×10^9^/L, a haemoglobin level of ≥90 g/L and <grade 2 non-hematology toxicity. Dose modification was according to the NCCN guideline and it was done by protocol, briefly, if more than grade 3 of non-hematology toxicity (except for nausea and vomiting) and grade 4 of hematology toxicity, neutropenia without febrile lasting more than 7 days, febrile neutropenia or infection and/or thrombocytopenia associated with bleeding occurs, the dose of the cytotoxic agents in the next cycle was reduced by 25%. Toxicities were followed up to 4 weeks after chemotherapy.

Clinical data obtained form patient’s charts. All patients were assessed with a complete medical history and underwent physical examination and laboratory analysis, including routine hematology and biochemistry analyses, TNM staging according to NCCN guideline, chest radiographs and computed tomography (CT) scan of the thorax and abdomen. Bone scan, CT scan or magnetic resonance imaging (MRI) scan was required under symptomatic indication. Hematology and biochemistry analyses were repeated every cycle or before chemotherapy for day 8 treatment. Toxicity was classified according to the common toxicity criteria for adverse events National Cancer Institute Common Toxicity Criteria (NCI-CTC; version 3.0) (CTCAE 3.0) at each cycle for each patient [13] and treatments of adverse events were supposed to follow CTCAE.

### Sample Collection and SNP Genotyping

Venous blood was collected from each subject into tubes containing 50 mmol/l of EDTA, at the time of enrollment, and genomic DNA was isolated with the QIAmp DNA blood Mini kit (Qiagen, Germany), according to the manufacturer’s instructions. Genotyping was performed by the AllGloTM probe (AlleLogic Biosciences, USA). The probes and primers were designed by using the Primer Express Oligo Design software v2.0. PCR reactions were performed in Lightcycler 3.0 (Roche, Germany). Data was analyzed by the Roche Lightcycler 3.0 software. Each reaction mixture of PCR (25 µl) contained 50 ng of DNA, 250 nM of each forward and reverse primer, 250 nM of each allele specific probe, and 12.5 µl of TaqMan Universal PCR Master Mix. Primer, probe sequences and amplification conditions are shown in Appendix A. For each SNP a minimum of 20 randomly selected DNA samples were genotyped at least twice to confirm the results. In addition, another set of random samples were taken to verify the results of SNP genotype by DNA sequencing (http://bioinfo.iconcologia.net/snpstats/start.htm). Polymorphisms were assessed using the AllGloTM probes (Chaoshi Biolology Company, Shanghai, Patent Number: 60/23263).

### Statistical Analyses

In order to compare the characteristics of the patient groups based on the six possible genotypes of ERCC5, ERCC6, MMS19L, CCNH, XPC and RRM1, chi-square test was used to ascertain differences in proportions between groups for the categorical variables. Logistic regression analysis was used to estimate the odds ratios (ORs) and 95% confidence intervals (CIs). Spearman correlation coefficient analysis was used to determine the correlation between different genotypes. The Hardy–Weinberg equilibrium assumption was assessed by the standard method of matching the observed numbers of individuals in the different genotype categories with those expected under Hardy–Weinberg equilibrium for the estimated allele frequency and comparing the Pearson goodness-of-fit statistic with the chi-square distribution with one degree of freedom. Genotype distributions were compared with the use of cross tabulation table analysis. For the evaluation of adverse events, patients experiencing all grade 3/4 adverse events were considered “responders”, and all other patients were considered “non-responders”. The response rates of patients according to genotype were compared in two separate comparisons by cross tabulation tables, evaluated by the chi-square test. Meanwile, multivariate analysis was done to find whether clinical characteristics, including age, gender, smoking, ECOG PS, histology, and chemotherapy regimens, influence the toxicity. Significance was set at 5% and all reported values are two-tailed. All analyses were performed with the SPSS software package, version 17.0 (SPSS Inc, Chicago, IL).

## Results

### Patient Characteristics

Totally 388 patients with cytologically or histologically confirmed NSCLC were recruited prospectively from October 2005 to March 2009. Given that the SNPs evaluated in the present study were in patients who received first-line chemotherapy. Finally, 365 patients were included in this study.

Baseline characteristics are shown in Table 1, All people are Asian. 33.2% had stage IIIB disease, and 66.8% had stage IV disease. Median age was 60 years (range 30–78), 68.8% were male, 36.2% were ECOG PS 1, 39.7% were diagnosed as squamous carcinoma and 7.9% as NOS-NSCLC. There were 169 never smokers (46.3%). No patient had received definitive thoracic radiotherapy, whereas 3.6% of the stage IIIB and 4.4% of the stage IV patients were radiated as palliative treatment to relieve symptoms caused by airway obstruction or pain by bone metastasis. The median follow-up time was 18 months (range 8–66 months) and 286 death events were recorded on the last date of May 2010.

**Table 1:** Characteristics of patients.

Characteristics	Number (%)
Median age (range)	60 (30–78)
Gender	
Male	251 (68.8)
Female	114 (31.2)
Race (% of patients)	Asian (100)
Smoking status	
Smokers	196 (53.7)
Non-smokers	169 (46.3)
Performance status	
0	132 (36.2)
1	233 (63.8)
Disease stage	
IIIb	121 (33.2)
IV	244 (66.8)
Histological type	
Squamous carcinoma	145 (39.7)
Adenocarcinoma	191 (52.3)
NOS-NSCLC	29 (7.9)
Chemotherapy regimens	
NP	67
NC	30
GP	171
GC	20
T/P+P	27
T/P+C	25
Non-platinum	25

* NP =  vinorelbine+cisplatin;

NC =  vinorelbine+carboplatin;

GP =  gemcitabine+cisplatin;

GC =  gemcitabine+carboplatin;

T/P+P =  docetaxel/paclitaxel+cisplatin;

T/P+C =  docetaxel/paclitaxel+carboplatin.

All patients had received the third generation combined chemotherapy, platinum-based or non-platinum-based. 97 received cisplatin/carboplatin plus vinorelbine (vinorelbine -cisplatin/vinorelbine –carboplatin, NP or NC), 191 had cisplatin/carboplatin plus gemcitabine regimens (gemcitabine-cisplatin/gemcitabine-carboplatin, GP or GC), and 52 were given cisplatin/carboplatin plus taxel/docetaxel regimens (docetaxel-cisplatin/docetaxel-carboplatin, DP or DC; taxel-cisplatin/taxel-carboplatin, PP or PC). The other 25 received non-platinum-based doublet chemotherapy with the third generation chemotherapy, such as gemcitabine-vinorelbine or gemcitabine-docetaxel. (table 1).

### Genotype Distribution

The allelic frequencies (ERCC5 D1104H, ERCC6 M1097V, ERCC6 Q1413R, ERCC6 R1213G, MMS19L G811A, CCNH V270A, XPC Q940K, XPC R500W and RRM1 C37A) and the distribution of all genotype information (wild-type, heterozygous and homozygous polymorphic variants) are listed in table 2. Genotype frequencies were consistent with previous reports and were in agreement with Hardy–Weinberg equilibrium model. Hematological toxicity was the most common adverse events.

**Table 2:** Allele frequencies of the gene polymorphisms in 365 patients treated with chemotherapy.

Gene	Mutation	Wild-type(N/%)	Heterozygous(N/%)	Homozygous(N/%)
**ERCC5**	D1104H, C/G	102(27.7)	181(49.2)	82(22.3)
**ERCC6**	M1097V,A/G	329(90.1)	27(7.1)	7(2.5)
	Q1413R,A/G	330(90.4)	35(9.6)	0
	R1213G, A/G	330(90.4)	35(9.6)	0
**CCNH**	V270A, T/C	307(84.1)	54(14.8)	4(1.1)
**MMS19L**	G811A, G/A	114(31.2)	182(49.9)	69(18.9)
**XPC**	Q940K, A/C	145(39.7)	173(47.4)	47(12.8)
	R500W, C/T	151(41.4)	157(43)	56(15.3)
**RRM1**	C37A, C/A	169(46.3)	170(46.6)	26(7.1)

N = Number of patients.

### Toxicity Related to SNPs

Patients received at least one chemotherapy cycle and were analyzed for toxicity. Toxicity profile and general adverse events distribution were shown in table 3. Grade 3/4 adverse events took place in 27.6% patients, totally. Only 2.7% patients did not suffer any toxicity. Among the hematological toxicity, leucopenia and neutropenia were the major adverse events, recorded in 63.3% and 41.9% of patients, respectively. Grade 3/4 neutropenia occurred in 12.3% of patients. Three cases of febrile neutropenia were recorded. Anemia and thrombocytopenia were observed in 49.9% and 25.2% of patients, respectively. Grade 3/4 anemia and thrombocytopenia were recorded in 1.9% and 5.7% of patients, respectively. The top 6 non-hematological toxicities were glutamate pyruvate transaminase increasing (41.4%), vomiting (24.9%), jaundice (23.3%), creatinine increasing (12.6%), infection (4.7%) and diarrhea (3.6%), respectively.

**Table 3:** General adverse events and distribution.

	0	Grade 1	Grade 2	Grade 3	Grade 4
**Hematologic toxicity**	(N/%)				
leukopenia	134(36.7)	103(28.2)	97(26.6)	27(7.4)	4(1.1)
neutropenia	212(58.1)	63(17.3)	45(12.3)	34(9.3)	11(3)
Anemia	183(50.1)	102(27.9)	73(20)	7(1.9)	0
thrombocytopenia	273(74.8)	34(9.3)	37(10.1)	14(3.8)	7(1.9)
**Non-hematologic toxicity**	(N/%)				
GPT increasing	214(58.6)	134(36.7)	11(3)	5(1.4)	1(0.3)
Cr increasing	319(87.4)	46(12.6)	0	0	0
diarrhea	352(96.4)	2(0.5)	11(3)	0	0
vomiting	274(75.1)	29(7.9)	55(15.1)	7(1.9)	0
jaundice	280(76.7)	55(15.1)	25(6.8)	4(1.1)	1(0.3)
infection	348(95.3)	2(0.5)	12(3.3)	1(0.3)	2(0.5)
**Total**	10(2.7)	90(24.7)	164(44.9)	76(20.8)	25(6.8)

GPT increasing: Glutamate pyruvate transaminase increasing Cr increasing: Creatinine increasing.

The correlation of SNPs in NER with general adverse events profile was listed in table 4. Table 5 showed Logistic regression analysis of relationship between NER genes and total grade adverse events or grade 3/4 adverse events.

**Table 4:** Relationship of SNPs in NER genes and general adverse events profile (%).

	0	Grade 1	Grade 2	Grade 3	Grade 4
**MMS19L(G811A)**					
AA	2	14	39	11	3
AG	5	51	65	48	13
GG	3	25	60	17	9
**ERCC6(R1213G)**					
AA	9	85	147	67	22
AG	1	5	17	9	3
**ERCC6(M1097V)**					
AA	10	84	146	67	22
AG	0	5	12	7	3
GG	0	1	6	2	0
**ERCC6(Q1413R)**					
AA	10	85	146	67	22
AG	0	5	18	9	3
**RRM1(C37A)**					
AA	1	7	10	7	1
AC	5	44	77	34	10
CC	4	39	77	35	14
**XPC(R500W)**					
CC	5	43	67	27	9
TC	4	34	71	35	13
TT	1	13	25	14	3
**XPC(Q940K)**					
AA	3	31	66	36	9
AC	5	47	75	32	14
CC	2	12	23	8	2
**CCNH(V270A)**					
CC	1	1	1	1	0
TC	1	13	29	9	2
TT	8	76	134	66	23
**ERCC5(D1104H)**					
CC	2	21	39	12	8
GC	7	45	75	44	10
GG	1	24	50	20	7

As shown in table 5, SNPs of MMS19L may have some roles in predicting the adverse events of chemotherapy in NSCLC, especially leucopenia (OR = 2.939, *P* = 0.020), jaundice (OR = 1.262, *P* = 0.037) and creatinine increasing (OR = 4.436, *P* = 0.013). In terms of grade 3/4 adverse events, SNPs of MMS19L and RRM1 have the highest predictive effect. SNPs of MMS19L were related with total grade 3/4 adverse events (OR = 1.091, *P* = 0.024) and grade 3/4 thrombocytopenia (OR = 1.119, *P* = 0.035), while SNPs of RRM1 were related with total grade 3/4 adverse events (OR = 5.159, *P* = 0.047) and with grade 3/4 vomiting (OR = 2.319, *P* = 0.046). ERCC5 was related with more infection (OR = 1.721, P = 0.017). No statistically significant association was found between the SNPs of ERCC6, XPC, CCNH and toxicity.

**Table 5:** Relationship between NER genes and general adverse events or grade 3/4 general adverse events profile (logistic regression).

MMS19L(G118A)			
	Odd ratio (OR)	95%CI	*P* value
All grade leukopenia	2.939	1.078–22.478	0.02
All grade jaundice	1.262	1.175–2.103	0.037
All grade creatinineincreasing	4.436	2.018–10.372	0.013
Grade 3/4 adverseevents	1.091	1.003–4.773	0.024
Grade 3/4thrombocytopenia	1.119	1.002–3.839	0.035
**RRM1(C37A)**			
Grade 3/4 adverseevents	5.159	1.025–25.971	0.047
Grade 3/4 vomiting	2.319	1.241–10.016	0.046
**ERCC5(D1104H)**			
Infection	1.721	1.002–13.215	0.017

There was no statistically significant association between the clinical characteristics and toxicity from the results of multivariate analysis by age (OR = 0.990, P = 0.357), gender (OR = 0.837, P = 0.464), smoking (OR = 0.662, P = 0.073), ECOG PS (OR = 0.760, P = 0.286), disease stage (OR = 1.027, P = 0.916), histology (OR = 1.454, P = 0.082), and chemotherapy regimens (OR = 0.875, P = 0.439) (Table 6).

**Table 6:** Multivariate analysis between the clinical characteristics and toxicity.

Characteristics	OR	95%CI	*P* value
Age	0.990	0.968–1.012	0.357
Gender	0.837	0.520–1.348	0.464
Smoking status	0.662	0.370–1.045	0.073
Performance status	0.760	0.459–1.258	0.286
Disease stage	1.027	0.624–1.689	0.916
Histological type	1.454	0.897–2.002	0.082
Chemotherapy regimens	0.875	0.623–1.228	0.439

## Discussion

In this study, we investigated whether SNPs in NER pathway genes were associated with toxicities of platinum-based chemotherapy treated in advanced NSCLC patients. We found that some SNPs in NER pathway genes were correlated with toxicity in advanced NSCLC patients, especially for SNPs of MMS19L, RRM1 and ERCC5 could have some role in predicting the adverse events of chemotherapy in NSCLC patients.

Standard treatment for NSCLC is platinum based doublet chemotherapy with third generation cytotoxic agent. None the less, one limitation of platinum-based chemotherapy is the unpredictable and occasionally significant side effects, including gastrointestinal and hematologic toxicity, which often complicate the clinical situation as it may impair the functional status of patients or their ability to tolerate further therapies. Platinum compounds form both intrastrand and interstrand DNA adducts that result in bulky distortion of DNA and destabilization of the double helix. Unless these adducts are repaired before the DNA replicates, they may lead to nucleotide substitutions, deletions, and chromosomere arrangements (mutagenesis) or to activation of cell signaling pathways that result in cell death [14]. These adducts are responsible for the cytotoxicity of the platinum agents, and clinical outcome seems to be related with the level of platinum-DNA adducts in the circulation [15]. Repair of DNA damage is a complex process carried out by a various array of DNA repair pathways, including NER and base excision repair (BER) pathways, and DNA mismatch repair (MMR) and so on [16]. Nucleotide excision repair is the major pathway for removing damaged bases from DNA. It suggests that suboptimal DNA repair actually may lead to the decreased removal of deleterious DNA lesions in normal bystander cells and therefore increase toxicity to platinum therapy. As such, constitutive variation in nucleotideexcision repair (NER) activity may be a prognostic factor for treatment-related toxicities in advanced NSCLC [17], [18].

ERCC5 might be involved in the efficacy of oxaliplatin containing chemotherapy for advanced colorectal cancer [19]. The inheritance of low-efficiency genotypes involved in DNA repair and replication may contribute to the difference in susceptibility of lung cancer. It was more frequent in patients with polycyclic aromatic hydrocarbon-DNA (PAH-DNA) adduct levels lower than the mean in NSCLC [20]. ERCC6 encodes a DNA-binding protein that is important in transcription-coupled excision repair. The protein appears to interact with several transcription and excision repair proteins, and may promote complex formation at repair sites. One case-control analysis revealed ERCC6 rs3793784:C>G alters its transcriptional activity and may confer personalized susceptibility to lung cancer [21]. MMS19 splice variants possess distinct functional domains, MMS19 exert its function in repair and transcription, specific MMS19 domains with distinct roles in NER and transcription and proposes the possible contribution of MMS19 protein isoforms in regulating the switch between transcription and NER [22]. Cyclin H (CCNH) belongs to the highly conserved cyclin family which functions as regulators of CDK kinases. Previous study shows that it also participates in the process of NER [23]. Xeroderma Pigmentosum group C (XPC), which is localized at 3p25 and encodes a protein of 940 amino acids that in vivo form a supramolecular complex, plays an important role in DNA repair. Defective XPC functioning has been shown to result in a cancer prone phenotype. More recently, Multiple in vivo and in vitro experiments indicate that XPC appears to be involved in the initiation of several DNA damage-induced cellular responses [24]. Ribonucleotide reductase subunit M1 (RRM1) is located on chromosome segment 11p15.5, which is required for deoxynucleotide production that is a crucial step in DNA synthesis and repair. Clinical studies suggest that overexpression of RRM1 is correlated with resistance to gemcitabine-based therapy [25].

SNPs in NER pathway genes might potentially be predictive factors for toxicity of cancer treatment. Sakano S et al [26] comprised of 101 bladder cancer patients treated with platinum-based chemoradiotherapy, and seven polymorphisms in XPC, XPD, XPG, XRCC1, XRCC3, TP53 and MDM2 were genotyped. They found More than two total variant alleles in nucleotide excision repair genes were significantly associated with grade 3/4 neutropenia. Any grade 3/4 hematological toxicity was significantly associated with the Gln/Gln or Lys/Gln + Gln/Gln genotypes of XPC compared with Lys/Lys. Kweekel DM et al [27] studied 91 advanced colorectal cancer to identify SNPs in DNA repair pathways that are associated with efficacy and toxicity in patients receiving oxaliplatin and capecitabine. They found the genesO-6-methylguanine-DNA methyltransferase (MGMT A), ligase I (LIG1) and ERCC2 were significantly associated with grades 3/4 toxicity. Carriers of the mutant allele showed a lower risk of developing grades 3/4 toxicity. Wu W et al [11] used matrix-assisted laser desorption/ionization time-of-flight mass spectrometry to genotype the three polymorphisms of XPD in 209 stage III and IV non-small-cell lung cancer patients treated with platinum-based chemotherapy. The variant homozygotes of XPD p.Arg156 Arg (rs238406) polymorphism were associated with a significantly increased risk of grade 3/4 hematologic toxicity, and, more specifically, severe leukopenia toxicity. Wang ZH et al [28] evaluated the predictability of DNA repair XRCC1 SNPs for cisplatin-based grades 3/4 chemotherapy-related toxicity in patients with newly diagnosed advanced lung cancer. They found that at least one variant XRCC1 Arg399 Glnallele was associated with a significantly increased risk of overall grade 3/4 toxicity and grade 3/4 gastrointestinal toxicity.

Our study have some limitation. Firstly, our study is a retrospective study and many factors, especially for different combination regimens, may influence results. Secondly, the incidence of chemotherapy-related toxicity in our study is relatively lower than other trials reported before, especially for grade 3/4 toxicity. The lower rate of toxicity may influence the results of our study. Thirdly, the relatively small number of patients in our study may be not have sufficient power to detect potentially relevant differences. However, most of these SNPs have not previously been studied whether they are related with toxicity induced by chemotherapy. This is first time to study whether these SNPs are related with toxicity. we still found some SNPs in NER pathway genes correlated with toxicity in advanced non-small-cell lung cancer patients treated with chemotherapy.

### Conclusion

Some SNPs in NER pathway genes correlated with toxicity in advanced non-small-cell lung cancer patients treated with chemotherapy, especially for SNPs of MMS19L, RRM1 and ERCC5, may have some effect to predict the adverse events of chemotherapy in NSCLC patients.
